# Adult Hippocampal Neurogenesis in Different Taxonomic Groups: Possible Functional Similarities and Striking Controversies

**DOI:** 10.3390/cells8020125

**Published:** 2019-02-05

**Authors:** Marcus Augusto-Oliveira, Gabriela P. F. Arrifano, João O. Malva, Maria Elena Crespo-Lopez

**Affiliations:** 1 Laboratory of Molecular Pharmacology, Institute of Biological Sciences, Federal University of Pará, Belém 66075-110, Brazil; marcusoliveira@globo.com (M.A.-O.); gabrielaarrifano@uol.com.br (G.P.F.A.); 2 Laboratory of Research on Neurodegeneration and Infection, University Hospital João de Barros Barreto, Federal University of Pará, Belém 66073-005, Brazil; 3 Laboratory of Experimental Neuropathology, Department of Pharmacology, University of Oxford, Oxford OX1 3QT, UK; 4 Coimbra Institute for Clinical and Biomedical Research (iCBR), and Center for Neuroscience and Cell Biology and Institute for Biomedical Imaging and Life Sciences (CNC.IBILI), Faculty of Medicine, University of Coimbra, Coimbra 3000-548, Portugal; jomalva@fmed.uc.pt

**Keywords:** neurogenesis, adult neurogenesis, species, memory, hippocampus, learning, brain, taxonomic level, human, cognition

## Abstract

Adult neurogenesis occurs in many species, from fish to mammals, with an apparent reduction in the number of both neurogenic zones and new neurons inserted into established circuits with increasing brain complexity. Although the absolute number of new neurons is high in some species, the ratio of these cells to those already existing in the circuit is low. Continuous replacement/addition plays a role in spatial navigation (migration) and other cognitive processes in birds and rodents, but none of the literature relates adult neurogenesis to spatial navigation and memory in primates and humans. Some models developed by computational neuroscience attribute a high weight to hippocampal adult neurogenesis in learning and memory processes, with greater relevance to pattern separation. In contrast to theories involving neurogenesis in cognitive processes, absence/rarity of neurogenesis in the hippocampus of primates and adult humans was recently suggested and is under intense debate. Although the learning process is supported by plasticity, the retention of memories requires a certain degree of consolidated circuitry structures, otherwise the consolidation process would be hampered. Here, we compare and discuss hippocampal adult neurogenesis in different species and the inherent paradoxical aspects.

## 1. Introduction

**Figure 1:**
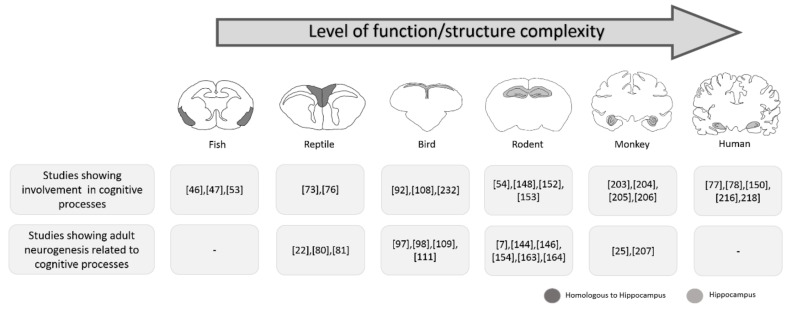
Schematic representation of hippocampal anatomic differences among species and studies showing involvement of these areas in cognitive processes (first line) and adult neurogenesis in these areas related to cognitive processes (second line). Although clear anatomical similarity only exists between the hippocampi of mammals, evidence points to a functional similarity with other species, such as fish or reptile, with less complex brain structure/function in general for cognitive processes such as memory, learning and future planning, among others (cognitive complexity). The birth of new neurons in adult mammals is mostly restricted to the hippocampal dentate gyrus. Although there are many neurogenic zones in other species, areas homologous to the hippocampus always appear among them, suggesting some similarity in the functions of these new neurons in cognitive processes in all species. Although a dense body of information points to similarities in function, it was suggested recently that the adult human brain does not retain the neurogenic capacity, raising intense debate about the possible impact on the way the human brain handles receiving, storing, and processing new information.

Adult neurogenesis was claimed by Joseph Altman in the early 1960s. After intracranial injection of [H^3^]-thymidine, an exogenous marker of cell proliferation, Altman found marked cells with the characteristics of neurons [1] that were able to differentiate from dividing precursors during adulthood. These new cells were added to the olfactory bulb and dentate gyrus [2]. Subsequently, the combined use of tritiated thymidine and electronic microscopy allowed the demonstration that these cells in the rat dentate gyrus presented neuronal characteristics, including dendrites and synapses [3]. The field later gained attention with new findings in birds [4] and mammals [5]. Although there are many differences between species regarding the hippocampal area and its homologues, a growing body of literature points to some important similarities (including those molecular, cellular, and functional), raising questions about the functional roles of adult neurogenesis in different species (Figure 1).

Mainly based on the later findings in rodents, adult neurogenesis is currently considered to play a relevant role in cognitive ability, especially in learning and memory. In this model, adult neurogenesis has been demonstrated extensively to increase with conditions improving cognition, such as enriched environment [6] and spontaneous physical activity [7,8]. Detrimental scenarios for cognition, such as stress [9], aging [10], neuroinflammation [11], and sleep deprivation [12], are also associated with reduced neurogenesis, at least in rodents.

A better approach to the phenomenon of adult neurogenesis has been possible with developments in technology and immunostaining techniques, the use of retrograde tracers, and ^14^C-labeling. This labeling methodology consists of the prior separation of cell nuclei immunolabeled for neuronal nuclei (NeuN), a mature neuronal marker, with subsequent analysis of DNA-^14^C, providing a precise estimation of the “age” of the neurons taking into consideration the exposure of people to environmental ^14^C released by nuclear bomb testing [13].

All of these advances have permitted the analysis of temporal dynamics, survival rates, and the circuitry integration preferentially adopted by new cells [14]. Moreover, the use of these tools to study several species with different neurogenic niches and different rates of neurogenesis helped us better understand this phenomenon.

Adult neurogenesis seems to be reduced, in regard to the number of both neurogenic zones and new neurons added into adult/established circuits, between fish and mammals. For example, up to 16 neurogenic zones can be detected in adult fish [15,16], but only six in rodents and three in humans [17,18]. The functions attributed to adult neurogenesis in the different species are related to their regenerative capacity, learning, and spatial, contextual, and emotional memories [19,20].

Interestingly, though adult neurogenesis is a well-established process in some species, in others, such as humans, it remains under intense debate. For example, the findings relating the generation of new neurons to hippocampal tasks, such as spatial ability and cognition, in adult hippocampal neurogenesis are contradictory. Adult neurogenesis is a recognized phenomenon in areas of the fish and reptile brains homologous to the mammalian hippocampus [21,22]. In addition, adult neurogenesis can be detected in the hippocampi of birds [23], rodents [14], nonhuman primates [24,25], and humans [26,27]. However, the absence or extremely low generation rate of new hippocampal neurons during adulthood has been demonstrated in bats [28], dolphins and whales [29], and recently even in humans [30], species that present a high spatial ability, with some occupying the top of the cognitive hierarchy.

Here, we review the knowledge regarding adult neurogenesis, highlighting its niches (especially the hippocampus) and possible implications on cognition across different species. We also discuss the recent conflicting findings regarding the presence or absence (or extreme rarity) of human adult hippocampal neurogenesis, raising methodological and conceptual issues.

## 2. Adult Neurogenesis in Fish

Fish have the highest number of neurogenic zones compared to other vertebrates [31], with up to 16 proliferative zones in some species [15,16,32]. In addition, more new cells are continuously generated in the fish central nervous system (CNS) than in other species, such as rodents [21,32,33,34].

The proliferative zones in the fish CNS include the olfactory bulb; dorsal telencephalon (dorsal zone, posterior zone, medial zone, and lateral zone), with the vast majority of cells found at the border between the dorsal zone and ventricle wall; and the preoptic area, hypothalamus, optic ceiling, cerebellum (four areas), and medulla (three areas) [21]. The widespread occurrence of this phenomenon may be closely associated with the high CNS regeneration capacity in these animals [33,35].

Some neurogenic zones, such as the dorsal telencephalon and hypothalamus, contain progenitor cells with radial glial characteristics, expressing glial fibrillary acidic protein, S100β protein, vimentin, and glutamine synthetase [36,37,38]. However, precursor cells that are negative for glial markers have been found in the dorsal telencephalon with unknown origin [37].

The rate of cell proliferation in the CNSs of these animals is also impressive. For example, brown ghost knifefish (*Apteronotus leptorhynchus*) and zebrafish (*Danio rerio*) are capable of generating an average of 100,000 new cells in 2 h and 6000 new cells in 30 min, respectively, corresponding to 0.2% and 0.06% of the total number of cells in the brain of each species [39]. In contrast, the dentate gyrus of the adult rat generates an average of 9000 cells per day, equivalent to only 0.003% of the total number of cells in the brain [40,41]. However, this proliferation is not homogenous, and it was recently shown that only granular cells in the cerebellum of zebrafish continue to be produced in adults and that only a few interneurons and glial cells are generated after 3 months of life [42].

Some areas in the fish brain are considered homologous to the hippocampus in mammals (Figure 1), including the lateral pallium of the goldfish (*Carassius auratus*) [43,44]. The goldfish pallium is essential for the association of discontinuous events over time [45], in addition to selective involvement in spatial memory [44], as has been demonstrated regarding the involvement of the dorsal lateral telencephalon in spatial learning tasks. Goldfish were trained in a spatial constancy task and had the activity of the three portions (dorsal, medial, and ventral) of the dorsal lateral telencephalon mapped for cytochrome oxidase. The ventral portion, but not the dorsal and medial portions, presented a significant increase in metabolic activity, suggesting that the ventral part is critically involved in spatial learning [46]. More recently, the same group investigated the dynamics of the involvement of the dorsal lateral telencephalon in a spatial leaning task and confirmed the involvement of only the ventral portion, besides demonstrating that this area acts like the dentate gyrus and *cornu ammonis* 3 (CA3) in mammals, performing separation and completion patterns [47].

In the zebrafish, the area related to the mammalian hippocampus is the dorsal lateral nucleus of the telencephalon [48]. Based on gene expression analysis, the dorsal subdivision of the pallium has been proposed to be homologous to hippocampal formation in the mouse [48]. Moreover, similar to rodents, zebrafish are able to perform simple and complex forms of associative learning, associating between cue and reward and between location and reward [49], as well as robust learning in a plus maze adapted from the mammalian literature [50]. Furthermore, the same physiological features of hippocampal sharp waves in rodents have been demonstrated in the anterodorsal nucleus of telencephalon, reinforcing homology [51]. More sophisticated functions, such as high-order representations of three-dimensional space, were already observed in pelagic fish [52].

Additional evidence has revealed a circuit in the pallium of the electric fish (*Apteronotus leptorhynchus*) between the dorsal pallium and dorsolateral pallium, similar to the hippocampal circuit in mammals [53]; this circuit can perform the same separation and completion pattern ascribed to the dentate gyrus and CA3 circuits, tasks that are closely associated with adult hippocampal neurogenesis in rodents [54].

Considering these anatomical and functional similarities, it would be very interesting to investigate the possible involvement of fish adult neurogenesis in these cognitive functions as a powerful tool to better understand the relationship between adult neurogenesis and cognition, especially because of the higher structural plasticity of the fish CNS compared to that of mammals [55]. Interestingly, transplantation of rat hippocampal progenitor cells into embryonic zebrafish recently showed that the developing zebrafish may be an efficient model for investigating the plasticity of several types of adult stem cells and external factors in cell fate [56].

## 3. Adult Neurogenesis in Reptiles

Adult neurogenesis in reptiles is well-established in both normal and post-injury conditions [57,58,59]. The generation of new neurons in the adult reptilian brain is a generalized phenomenon found in the four divisions of the cortex, the main and accessory olfactory bulbs, the rostral forebrain, the septum, the striatum, the *nucleus sphericus*, and the anterior dorsal ventricular ridge [58,59,60,61,62,63,64].

Most studies of adult neurogenesis in reptiles have focused on lizards and turtles and used a combination of neuronal markers and bromodeoxyuridine (BrdU), a thymidine substitute in DNA synthesis that is incorporated during the S-phase of the cell cycle [57,58,65]. In these studies, cell proliferation was mainly detected in the ependymal layer of the lateral ventricles [65]. Moreover, three zones (olfactory system, telencephalon, and cerebellar cortex) suggestive of neurogenesis were recently revealed in the Nile crocodile (*Crocodylus niloticus*) by immunostaining doublecortin (DCX), a microtubule-associated protein involved in the migration of new neurons [66]. However, adult neurogenesis may occur mainly in the telencephalon, and more extensively in the medial cortex [67].

The medial cortex of the lizard is a trilaminar region that exhibits many characteristics similar to the mammalian dentate gyrus, as demonstrated by the Golgi impregnation method and immunocytochemistry studies [68,69]. The neuronal types of the dorsomedial cortex of reptiles, the parahippocampal area in birds, and the CA3 region in mammals may present with homology [70] (Figure 1). Supporting the hypothesis of homology between these regions, a recent study confirmed the existence of spatial memory in side-blotched lizards (*Uta stansburiana*) using a Barnes maze, a spatial memory test widely used in mammals [71].

Interestingly, the medial cortices in the turtle and lizard express the mammalian pan-hippocampal transcription factor ZBTB20. Furthermore, in turtles, PROX1 and MEF2C, which are found in mouse dentate gyrus granular cells, have been demonstrated to be labeled in the medial cortex, and ETV1, MEIS2, and LMO4, which are found in the mouse CA, have been demonstrated to be labeled in the dorsomedial cortex, suggesting dentate gyrus–CA-like neurons in reptiles [72]. Unbiased analysis of the cell-type transcriptome has demonstrated significant overlap between the mouse dentate gyrus and turtle medial cortex and between the mouse CA and turtle medial cortex. These areas share several genes encoding K^+^ channel subunit proteins, cadherins (CDH8) involved in the formation of dentate gyrus–CA3 synapses, and LRRTM4 and CNIH3, which are involved in synaptic transmission [72].

Injury to the medial cortex of the turtle compromises spatial memory task performance [73], just as hippocampal lesions in birds [74] and mammals [75] impair spatial performance in these animals. Moreover, the medial cortex of rattlesnakes increases in size with increasing navigational demand [76], as has been demonstrated to occur with the human hippocampus [77,78]. Investigating the possible interaction between adult neurogenesis and territoriality and environment, male lizards accommodated in larger environments exhibit higher neurogenesis in the medial cortex than animals living in smaller environments [22]. Recently, doublecortin positive (DCX+) neurons were shown to be highly numerous in the medial cortex of the lizards, particularly in the granular cell layer, though they are scarce in the dorsomedial cortex [79].

In turtles, a deep water enriched environment providing opportunities for increased physical activity results in increased neurogenesis compared to the animals deprived of such stimuli [80]. In agreement with these findings, neurogenesis is decreased in the Tenerife Lizard (*Gallotia galloti*) in captivity compared to wild animals [81].

Considering the functional homologies between the reptile cortex, parahippocampal area of birds, and the hippocampus of mammals, the level of adult neurogenesis in reptiles probably correlates positively with learning and memory rates, even influencing these cognitive processes to some extent. Future studies are required to demonstrate the validity of this hypothesis and will permit a better understanding of the possible relationship between adult neurogenesis and cognition, as in the case of fish.

## 4. Adult Neurogenesis in Birds

Adult neurogenesis in birds was investigated for the first time about a decade after its discovery in rats. The generation of neurons in the adult bird brain was demonstrated in the ventricular hyperestriatum using [H^3^]-thymidine [4]. Since then, several studies have revealed the existence of adult neurogenesis as a well-established and widespread phenomenon in birds [82,83,84,85,86].

Although the proliferation, migration, differentiation, and insertion of newborn neurons in the circuits of the adult bird brain still need to be investigated, we have some knowledge about this complex process. The proliferation of precursor cells that will become future neurons is found mainly along the ventrolateral and dorsomedial ventricular wall zone. These cells account for 93% and 6% of the total number of proliferative cells in the ventrolateral and dorsomedial ventricular wall zone, respectively, and were identified as the radial glia, which divide and assume the identity of young neurons [87]. These findings were confirmed posteriorly by ultrastructural analysis [88]. In other areas such as the high vocal center (HVC), approximately 30% of new neurons are associated with radial glia [89].

A recent study in parrots (*Psittacus erithacus* and *Psittacus timneh*), using a marker of cell proliferation, the Proliferating Cell Nuclear Antigen (PCNA), and DCX, confirmed that neurogenesis is scattered in the adult brain [90]. New cells were found in the olfactory bulbs, diencephalon, rhombencephalon, the subventricular zone of the lateral wall of the lateral ventricles, and the telencephalic subdivisions of pallium and subpallium. These areas comprise hippocampal formation, an area intimately involved with learning and memory processes, suggesting a role of neurogenesis in circuit plasticity during learning and adaptation to the environment [90].

New neurons take 7 to 11 days to migrate from the ventricular zone to their target areas in the brain where they differentiate into different neuronal phenotypes [91]. For example, 7 days after cell division, cells generated in the ventricular zone can be found in the hippocampus differentiated into new neurons [92]. 

From a quantitative point of view, the incorporation of new neurons varies among brain areas and species. For example, in the HVC, daily incorporation of newborn neurons fluctuates between 0.1% and 0.74% of the total neuronal population in canaries (*Serinus canaria domestica*), 0.1–0.2% in the zebra finch (*Taeniopygia guttata*), and approximately 0.4% in the Bengalese finch (*Lonchura striata domestica*). In the hippocampus, the incorporation of newborn neurons per day ranges from 0.15 to 0.37% of the whole population of hippocampal neurons [93].

The generation and survival of new neurons is influenced by several life-related factors, such as food restriction [94], seasonality [95], migratory experiences [86], migratory distance [96], and learning and memory [97,98].

Adult neurogenesis is associated with different functions. In some birds, such as canaries, it is closely related to mating singing control [99]. A paradoxical result has been found in zebra finch, in which singing capacities do not exhibit great changes when the number of neurons doubles [100]. In other birds, adult neurogenesis is associated with learning and memory processes [97,98]. Interestingly, induction of stress by extreme food restriction negatively influences hippocampal neurogenesis in chickens [94], suggesting similarities in the response of bird and rodent hippocampi to stressors.

There is an ongoing debate about the possible analogies between bird and mammalian hippocampi [101,102], but other than their clear functional homologies, strong evidence of neural circuit homology has already been shown. For example, studies using genetic expression [103,104,105,106], anatomical connectivity [107], and retrograde tracers [106] have extensively demonstrated analogies between the hippocampi of birds and mammals. In birds, the centers of high cell proliferation, called "hot spots", are found in the dorsal and medial lateral walls of the lateral ventricles [87]. These areas are involved in hippocampal formation and are intimately involved in cognition processes.

Accordingly, most experienced pigeons (in terms of migration) have increased neurogenesis in the hippocampus compared to less experienced pigeons, suggesting a relationship with spatial navigational experience and potentially expanded spatial memory [23], as we know that experienced birds use neural mechanisms to guide their journey and the hippocampal formation plays a central role in it [108]. Previously, spatial learning was demonstrated to induce neurogenesis in marsh tits [98]. New evidence recently emerged that links neurogenesis to spatial learning, as in the black-capped chickadee, in which inhibition of cell proliferation in the hippocampus impairs spatial memory [109].

Birds exhibit a strong ability to separate patterns. Some species that store food can discern hundreds of different cache locations, demonstrating a great ability to disambiguate spatial memories [110]. This ability seems to be affected by neurogenesis. Increased mistakes are detected in memory reversal test when hippocampal neurogenesis is reduced by methylazoximethanol acetate, supporting the possible relationship between adult hippocampal neurogenesis and separate patterns [111].

## 5. Adult Neurogenesis in Rodents

Since it gained strength roughly two decades after its discovery by Altman, adult neurogenesis in rodents has been the most investigated in animals. Therefore, much knowledge is available on the composition of rodent neurogenic niches, the modulation of their neurogenic cascade, and the role of adult newborn neurons in rodent brain function.

In vitro experiments and results in neonatal rodents initially pointed to two neurogenic zones in the rodent CNS, the subgranular zone (SGZ) of the dentate gyrus and subventricular zone (SVZ) of the lateral ventricles, and the hypothalamic neurogenic niche [112,113,114]. The generation/migration of neuroblasts in the adult brain has also been described in other areas of the rodent's brain, though not clearly confirmed, such as the amygdala [115], basal forebrain [116], neocortex [117], and substantia nigra [118].

Adult neurogenesis comprises proliferation, migration, differentiation, and incorporation of new cells into the functional neural circuits. Neurons born in adults take much longer to complete their maturation process compared to neurons that were born in the postnatal period, for example. The generation of new neurons from proliferating cells lasts about a month, and their complete differentiation into fully mature neurons may take several months, as the formation and maturation of synaptic spines continues for more than 2 months after their generation [119]. In contrast, in nonhuman primates, complete maturation time can take 6 months [120]. Between 2 and 6 weeks of age, these cells exhibit enhanced synaptic plasticity compared to older mature neurons, suggesting that new neurons present high probability of depriving mature cells of some synaptic inputs by competition during the 2–6-week time window [121], contributing to activity-dependent rewiring of the dentate gyrus [122]. During the maturation process, dendritic spine plasticity is present 4–5 weeks after cell birth [123]. Adult neurogenesis in mice accounts for a turnover of roughly 10% of hippocampal neurons in adulthood [124,125]. This number is higher in rats than in mice [126]. The functional representation of the neurons generated in some of these areas of the adult brain remains under debate. Neurons generated in the adult mouse amygdala, for example, are not affected by contextual fear conditioning [115]. Evidence suggests that adult neurogenesis in the hypothalamus is involved in adaptive functions developed to respond to changes in diet [127]. In the olfactory bulb, ablation of neurogenesis results in various olfaction-related defects [128,129]. Although knowledge is advancing towards a better understanding of the role of adult neurogenesis in these areas, it is important to note that the functional relevance of neurogenesis occurring in the dentate gyrus is by far the most extensively documented process.

Adult hippocampal neurogenesis in rodents does not play merely a regenerative role (i.e., a role in providing a substrate when cells are lost by aging, injury, or disease, for example, without a clear role related to cognition processes). The newly generated cells migrate, differentiate, and integrate into circuits [14], actively responding to environmental and internal stimuli [130]. For example, the neurogenic rate decreases with age in both the SGZ and SVZ [131], declining with cognitive function [10], with an enriched environment [6] and physical activity [8] preventing cognitive decline. Whether the age-related reduction in the number of new neurons is caused by a decrease in the number of neural precursors [132] or by increased quiescence of neural stem cells is unclear [133]. The continuous depletion of neural stem cells was recently shown to decrease hippocampal neurogenesis [134]. In addition, adult neurogenesis is affected by alcohol intake [135], inflammatory cytokines [136], physical activity [137,138], aging [139], and learning [140]. Adult neurogenesis also plays an important and well-documented role in fine learning and memory processes [141].

The use of retrograde tracers mediated by rabies virus has revealed that newly generated neurons in the SGZ of the dentate gyrus receive afferents from the entorhinal cortex increasing significantly with the maturation of cells over time. This innervation from the entorhinal cortex is stronger from the lateral entorhinal cortex than the medial entorhinal cortex [123,142], allowing a better understanding of the role of new neurons in cognition. These findings point to greater imputation of the lateral entorhinal cortex than the medial entorhinal cortex for newly generated granular cells, strongly suggesting a predominant involvement of these new neurons in tasks integrating new spatial information [14,123,142,143].

Voluntary running was recently shown to be able to improve spatial pattern separation in mice [144,145]. This is possibly due to increased neurogenesis in response to physical activity [144,145]. In addition, neurogenesis plays a crucial role in discriminating characteristics within events [146]. Adult neurogenesis modifies the excitatory circuit of the dentate gyrus; thus, adult newborn neurons may interfere and play a role in the cognitive functions attributed to this region of the hippocampus, such as pattern separation [147]. The role of modulating pattern separation [148], which discriminates environmental similarities by non-superimposed neuronal groups [149,150,151], is usually attributed to the dentate gyrus [148]. Several computational works have suggested that a certain degree of input separation is required for coding memories in CA3 [54,152,153]. 

To test the hypothesis that neurogenesis is necessary for discriminating between two stimuli with few differences between them, Clelland and colleagues used two types of spatial tasks, one navigational and one non-navigational. Eliminating local neurogenesis in the hippocampus of adult mice by applying low doses of X-irradiation, this group demonstrated that the animals had impaired performance when the stimuli were similar, but not when the stimuli were more different [154].

**Figure 2:**
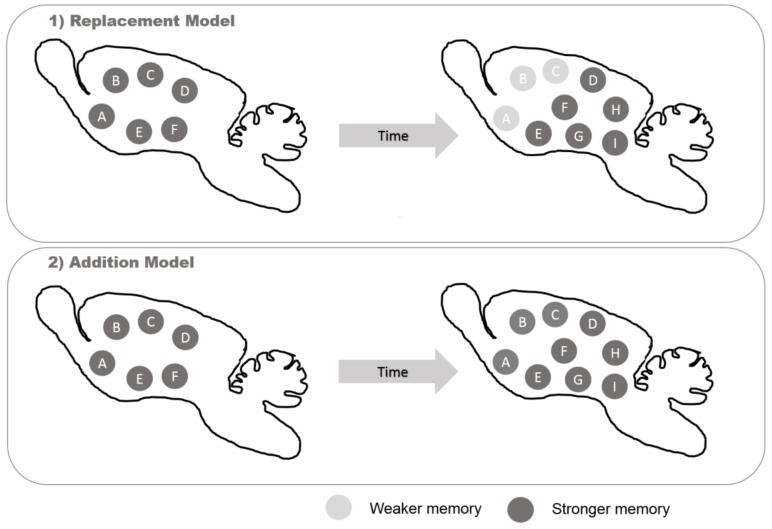
Examples of neurogenic models. The “replacement model” would replace dead neurons and prioritize new memories because older memories would be lost as older neurons lose their synaptic connection and are replaced by new ones. The “addition model” would prioritize more hard networks as newborn neurons are constantly added to the circuit and retain older memories. Although both models exist simultaneously, the two models and their possible consequences are investigated as some predominance of one or the other.

Several computational models have been generated to try to understand the contribution of adult newborn neurons to the general function of the dentate gyrus. The main difference in neurogenic models is between the substitution model and addition model (Figure 2). In the substitution model, new neurons replace the old granular cells, making new synapses. On one hand, this improves the learning process by enhancing synaptic flexibility [155]; on the other hand, this phenomenon is accompanied by forgetting older memories with loss of the information coded by the removed cells, a concern with adult neurogenesis known as the “stability–plasticity dilemma” [156]. For example, reduced neurogenesis is associated with reduced clearance of hippocampal memory traces [157]. In the addition model, new granular cells are mainly incorporated into the circuit, creating new synapses and allowing the old cells to maintain their connections. This process occurs with a constant addition of cells, considering the entire hippocampal–entorhinal loop (entorhinal cortex–dentate gyrus–CA3–CA1–entorhinal cortex), suggesting the maintenance of older memories for a longer time and a constant increase in the total number of dentate gyrus granule cells over time [158,159] (Figure 2).

Although the evidence of the involvement of adult neurogenesis in cognitive processes in rodents is substantial, there is also a considerable body of evidence indicating the opposite. For example, learning in the Morris water maze induced plastic changes in the dentate gyrus of adult rats unrelated to neurogenesis [160]. Though stress and aging are associated with reduced hippocampal neurogenesis [161], it does not necessarily translate into learning and memory deficits [162]. In addition, though some studies have demonstrated a positive correlation between learning and adult neurogenesis [7,163,164], other reports suggested that learning is associated with no change, or even a decrease, in the number of new neurons [165,166,167,168]. All of these findings suggest that the performance in different behavioral tests may be associated with adult neurogenesis to a different extent, from no relation to being highly dependent on the incorporation of new neurons into the hippocampal circuitry, and also depending on the status of brain parenchyma, such as healthy or under neuroinflammatory conditions.

Moreover, the ablation of mature granular cells, but not the ablation of young granular cells, interferes with spatial learning [169]. These adult-born mature neurons undergo profound somatic and dendritic modification because of spatial learning, suggesting that even adult-born mature cells may provide additional plasticity to the circuit [169]. Subsequently, both mature and immature granular cells may be recruited equally to spatial memory coding and retrieval [170]. Intense exercise has been shown to promote adult neurogenesis, but not spatial discrimination, suggesting an alternative mechanism for spatial memory rather than just an increase in the number of neurons [171].

Even with relevant advances in the field of adult neurogenesis, especially in rodents, careful and thorough investigation is still needed to unravel the precise functional roles of the continuous generation of neurons in such an important area for cognition. Several problems make our understanding of the role of adult neurogenesis in cognition difficult, as there are methodological limitations and issues related to the specificity of some frequently used markers. For example, adult hippocampal neurogenesis can be detected in DCX-knockout animals [172], though DCX is expressed by neurons and glia [173]. Moreover, BrdU can be incorporated into cells under stress, trying to cope with DNA repair [172,174,175]. Another important issue is the appropriate choice of behavioral tests that are really related to the functioning of young neurons, considering that neurogenesis may be associated with some, but not all, aspects of memory in the hippocampus [176]. Adopting appropriate and thoughtful immunohistological and behavioral protocols is a critical step in avoiding misleading conclusions.

## 6. Adult Neurogenesis in Nonhuman Primates

In nonhuman primates, adult neurogenesis was first documented using BrdU immunolabeling, demonstrating the existence of new neurons in the hippocampus of old-world primates [24,177]. In addition, other studies found newly generated neurons in other areas, such as the amygdala or neocortex [178,179].

Some differences and similarities can be highlighted in the process of adult neurogenesis in rodents and primates. In rodents, roughly 9000 neurons are inserted into dentate gyrus circuits per day [40], whereas only 1300 are inserted into the monkey dentate gyrus in the same temporal window [180]. The peak of postnatal neurogenesis in the monkey dentate gyrus is estimated to occur around 3 months after birth, and it is followed by a persistent intermediate level until 1 year of age, persisting again at a lower but significant level in the mature dentate gyrus [180]. In comparison, in rats, the generation of new neurons is stable at 4–7.5 months of age and subsequently decreases from 7.5 to 12 months of age, with a lower proliferation of neural stem cells underlining this decrease [181]. The neuronal maturation process in rodents takes about 2 months [182], whereas in the primate it takes 3 to 6 months [120,183]. Despite the different periods of maturation, the generation of new neurons declines with aging in both species [184,185]. Nonhuman primates have a much longer life span than rodents, and a much longer new neuron maturation period; whether a parallel exists between these dynamics and physiological and behavioral features in both species would be interesting to study. Perhaps different species can deal with this balance between maturation time, amount of proliferation, and survival ratio to supply their cognition demands in different ways, considering the differences in the hippocampal dentate gyrus among species [186], even though an imaging study had demonstrated that the dentate gyrus is the hippocampal subregion most affected by aging in both species [187].

Evidence in rodents, primates, and humans suggest that the temporal area is central to cognition [188,189,190,191] and that the hippocampus is related to the object, spatial, temporal, and contextual associations [192,193,194]. Interestingly, conserved molecular signatures in neurogenesis between rhesus monkeys and mice were found in genome-wide transcriptional analysis of the SGZ of the dentate gyrus [195].

Similar to findings in rodents [196], early-life stress alters metabolic parameters such as blood glucose levels, lipid ratios, and body weight [197], but it also reduces neurogenesis in young monkeys [198], as well as social isolation [199,200] and alcohol consumption [201]. These findings suggest that hippocampal neurogenesis of both species responds in a physiologically similar manner to negative stimuli.

Although anatomical and functional similarities have been demonstrated between the hippocampi of rodents and primates, the generation of new neurons in the hippocampus of adult monkeys is only modestly associated with cognitive disturbances [25], differing from what occurs in rodents, in which the participation of adult neurogenesis in cognitive processes is well-established [141].

Despite the molecular [195], cellular, and functional [202] similarities between rodent and primate hippocampi, our understanding of the potential functionalities of adult hippocampal neurogenesis in nonhuman primates needs to be advanced. Surprisingly, although evidence suggests hippocampal involvement in object-recognition memory [203], contextual memory [204], spatial memory [205], and pattern separation in nonhuman primates [206], no data definitively confirm the relationship between adult neurogenesis and cognition, only showing modest positive correlations [25,207].

## 7. Adult Neurogenesis in Humans

In the late 1990s, Eriksson and colleagues first described the ability of the adult human brain to generate new neurons. Using brains from human patients treated with BrdU and subsequently immunolabeled against BrdU and NeuN, enolase, or calbindin, they demonstrated that the adult hippocampus retains neurogenic capacity [208]. More recently, it was demonstrated using ^14^C dating that the human brain generates new neurons throughout adulthood, suggesting that adult hippocampal neurogenesis may contribute to brain function [27]. Despite studies describing no neurogenesis in areas such as the SVZ or hippocampus [30,209], the generation of new neurons has been demonstrated in the striatum [210] (with possible implications in psychiatric disorders [211]), lateral ventricular wall [212,213], and, to a limited extent, in the olfactory bulb [214].

In the adult human dentate gyrus, the daily turnover is 0.004%, lower than that of adult mice (0.03–0.06%) and adult monkeys (0.02%) [24,27,40,180]. The qualitative and quantitative changes in age-related hippocampal adult neurogenesis share similar characteristics with murine models [215].

Considering the assumed role of the dentate gyrus in pattern separation and cognition in rodents [54,154] and primates [206] and studies using high-resolution magnetic resonance imaging in humans that demonstrated a role of the dentate gyrus/CA3 regions in the pattern of separation [150,216,217,218,219], it seems reasonable that the generation of new neurons has similar functions in humans. However, the growing complexity of the brain has possibly changed the tools for dealing with some aspects of cognition.

Recently, severe controversies have shaken the field of human adult neurogenesis, challenging the findings and speculations. Using DCX, transcription factor SRY (sex determining region Y)-box 2 (SOX2), nuclear protein KI-67, and polysialic acid - neuronal cell adhesion molecule (PSA-NCAM), a group led by Alvarez-Buylla demonstrated that neurogenic levels decline to undetectable levels in the human hippocampus by 13 years of age, and it is extremely rare for the generation of new neurons to continue [30]. These findings are similar to the lack of new neurons in the adult brains of dolphins, porpoises, and whales [29], mammals with long lives and complex behaviors, suggesting high cognitive indexes. We must keep in mind that these animals inhabit very different environments from humans, with possible differences and similarities between the available cognitive stimuli remaining to be investigated. Although these cetaceans do not exhibit convolutions characteristic of the mammalian dentate gyrus, the absence of new neurons in adult specimens suggests some similarities with the human cognition process.

Also, recently, Boldrini and colleagues demonstrated that although angiogenesis and neuroplasticity decrease in the elderly, neurogenesis levels in the gyrus remain unchanged with aging [26]. This finding may support the hypothesis that adult neurogenesis is essential for specific cognitive functions in humans and that its decline is linked to compromised emotional-cognitive resilience.

Although markers, such as DCX immunolabeling, work when performed in control, neonatal brains [220], studies that do not find continuous neurogenesis in the human hippocampus discuss possible methodological difficulties, suggesting that new neurons continue to be generated in the hippocampus of adult humans. On the one hand, the lack of neuronal specificity in DCX immunolabeling, as it is also expressed by glial cells, may be responsible for false positives. On the other hand, DCX was one of the markers used by Sorrells and colleagues and shows an acute reduction in expression a few hours after death [221], possibly indicating a false negative effect. Boldrini's group examined brains within 26 h after death, almost half the postmortem time elapsed in Sorrells’ groups’ study. In addition, labeling with Ki-67, a cell proliferation protein expressed during mitosis and absent in quiescent cells, can be misinterpreted as changes associated with glia and vasculature rather than neurons [221]. Interestingly, Ki-67 and DCX labeling can also be influenced by the genetic background of different lineages [222]. Several limitations exist in the main methodologies employed in the study of adult neurogenesis [172,173,174,175].

Sorrells´ group also showed that DCX and PSA-NCAM only detect immature neurons accurately if both are expressed in the same cell, labeling both mature neurons and non-neuronal cells in human brain. They showed the possibility of immunohistochemically staining BrdU-like elements in tissues where BrdU is absent.

Excellent reviews have raised several points considering recent evidence supporting the lack of newly generated neurons in the adult brain [220,223,224,225,226]. The authors raised some points in common, such as postmortem delay, with great potential for misinterpretation. Another point is the absence of a stereological quantification of cells, a method established as the gold standard in histological studies and implemented by Boldrini’s group, which may be responsible for at least part of the difference found between the two studies. Stereological quantification is considered a gold standard because it is a very precise way to estimate the cell population from 3D tissue through algorithms that gives us the possibility of correcting several problems caused by fixation, cutting, and immunohistochemistry processes, i.e., contraction (uniform and nonuniform), cap loss, and different levels of cell density between the border and middle of tissue. The importance of a stereological approach is well raised by Kempermann and colleagues [223].

Another important point that rests on specific features of the samples used by Sorrells’ group is the control for postmortem delay. The resected samples present specific characteristics because of early seizures and antiepileptic drug administration, which in rodents are known to affect hippocampal circuitry and homeostasis. Moreover, DCX+ and PSA-NCAM+ cells have been found in the brains of young children, and often present different autofluorescent signals (lower than adult brains) and different water and glia content. In addition, children often die in the hospitals, reducing the postmortem delay, making it difficult to perform a direct comparison [224].

It is hypothesized that DCX antibody works better in animals. Considering this, Boldrini’s group performed double immunohistochemistry for DCX and neurofilaments (NFs) to try to identify DCX+ cell dendrites that are not immunolabeled. They found DCX+/NF+ cells with elongated morphology and dendrites extending through the granular cell layer, with migration morphology in the adult human SGZ [26]. Several technical issues remain to be optimized to make the human brain more accessible and investigate adult neurogenesis in depth. 

The rarity, or even lack, of adult neurogenesis in the hippocampus of nonhuman and human primates has significant implications for the function of adult neurogenesis, and even for a possible special function attributed to the mammalian dentate gyrus. The possible absence of adult neurogenesis in humans would provide a completely new perspective from which to think about how our brain operates cognitive processes, how it reaches the plasticity level for learning processes, and how it deals with new information and forgetting processes. This raises new questions about the functions of the dentate gyrus. One hypothesis suggests a special role of adult neurogenesis in the hippocampus considering the late specialization of the hippocampal dentate gyrus. This phenomenon may be a tool used by species with higher cognitive capacity, and therefore, would use the generation of new neurons in this region to deal with the complex cognitive adaptations required by these species [227].

Alternatively, clusters of non-newly generated DCX+ cells were recently demonstrated to be “trapped” in the white matter with no direct contact with neurogenic zones in Cetartiodactyla (*Tursiops truncates, Stenella coeruloalba,* and *Ovis aires*) [228] and in the whole adult sheep cortex [229]. As DCX+ cells maintain immature features, these findings show that these mammals (large-brained and relatively long-living mammals, similar to humans), unlike rodents, present large numbers of these non-newly generated immature neurons, suggesting that they may use “young neurons” more than new neurons to deal with plastic needs, an alternative method compared to the prevalent neurogenic view.

However, it is intriguing that a region presenting so many strong similarities between rodents and humans uses so many different ways of dealing with cognitive demands. Another interesting point is that humans with an “ever-young” dentate gyrus, as hippocampal neurogenesis presents only a modest reduction [27]—if any—with age [26], have a remarkable cognitive decline related to aging, even with normal aging. The fact that neuroplasticity undergoes an age-dependent decline and neurogenesis does not would entail profound changes in how we think about functions strongly attributed to the new neurons.

Brain complexity suggests that increased area results in increased functionality [230], and in some cases, the proportion of new neurons follow this phenomenon. For example, compared to rodents, a reduced olfactory ability in humans correlates with a reduced size and the rarity of adult neurogenesis in this region [214], in addition to no substantial migration of neuronal precursor cells (NPCs), called neuroblasts, from the subventricular zone to the olfactory bulb [213]. Similarly, the striatum is a phylogenetically new area, enlarged parallel to the neocortex with the growing complexity of the brain. Humans exhibit a very pronounced striatal adult neurogenesis [17] compared to rodents [117] and monkeys [231], perhaps related to the improvement of emotional, cognitive, and movement skills. Curiously, this parallel fails with the hippocampus. The dentate gyrus of the hippocampus, a center of cognitive processing, is associated with spatial ability across species [232]. In humans, the occurrence of strong and even permanent adult neurogenesis [26,27] or extreme lack of generation of new neurons in the adult hippocampus [30] is currently under intense debate, highlighting the need for innovative approaches to study neurogenesis in adult humans.

Some arguments, such as nonlinearity in the relationship between the rate of new neurons and their functional relevance, which circumvents the lowest neurogenic rate in humans, encounter serious difficulties, with the possible lack or extreme rarity of new neurons in the adult brains of some species occupying the top of the ranking in terms of CNS complexity. On the other hand, considering the large interindividual neurogenic variations demonstrated with diverse markers [27,233], it is possible that the individuals in the sample in Sorrells’ study presented with the minimum levels of generation of new neurons. It is also important to note the absence of stereological quantification, a method established as the gold standard with correct identification in histological studies and implemented by Boldrini’s group, which may be responsible for at least part of the difference between the two studies.

Other possibility is that the higher complexity of brain structure/function has prioritized less drastic and more metabolically economic methods for dealing with the plasticity required for learning and memory. Although adult neurogenesis may be considered a solution for the plastic demands associated with cognition, the possible deleterious effects of adult neurogenesis have been demonstrated. The continuous generation of new neurons generates oxidative stress [234], as well as changes in the local environment because of the possible addition of 700 new neurons per day in each hippocampus [27], which may increase the likelihood of brain tumors; some studies suggest a relationship between defective neural stem cell differentiation and the emergence of glial tumors [235]. Furthermore, neurogenesis improves the learning of new information, but it is also associated with the forgetting of old memories. Greater flexibility in neural circuits has been associated with low memory retention, whereas less flexibility has been associated with greater memory retention [236].

To generate new cells, including the processes of division, migration, and differentiation before insertion into pre-existing circuits, there is a very complex metabolic demand that the human brain must achieve in both the acute (e.g., when adult neurogenesis is enhanced by some acute behavior or physical activity) and chronic (e.g., when new cells are born without the impulse from the elements cited above) neurogenic processes [237,238].

A possible alternative to the constant addition of new neurons to the adult hippocampal circuit could be the turnover of dendritic spines, especially considering the well-established involvement of synaptic plasticity by dendritic remodeling in cognitive processes related to learning and memory [239,240], in which the turnover of dendritic spines continues even after the connections are formed [241]. These events may cover the cognitive necessities of adapting to the most diverse environments and situations in a more comprehensive manner than previously thought. With the improvements in existing methods and the emergence of new tools, many of these questions will be answered more accurately in the near future.

## 8. Conclusions

Adult neurogenesis has been shown to occur in many species from fish to mammals, with the generation of new neurons in the hippocampus and homologous areas pointing to functional similarities at all taxonomic levels. Although there are some discrepancies, the neurogenic rate in general correlates in the same direction with positive factors such as physical activity, environmental enrichment, and learning and negative factors such as aging, neuroinflammation, and stress in all species studied thus far.

In humans, which have the greatest CNS complexity, the findings are strongly contradictory to the generation of new hippocampal neurons in adults, and data do not yet exist on the function of these new cells due to the lack of tools to detect adult neurogenesis in living humans. In addition, some methodological differences in the analysis of postmortem human samples, especially regarding the specificity of the cell markers used, the postmortem delay to adequately preserve the tissues, and the cell counting systems employed, may account for much of the discrepancy observed when describing the existence of adult neurogenesis in humans.

This review contributes to the compilation of what we presently know about hippocampal adult neurogenesis across species, highlighting its function related to retrograde and anterograde memory processes. Major questions are raised about how we deal with incoming information and pre-existing information in the hippocampal formation circuitry. More research and the development of new techniques are needed to more accurately determine whether new neurons continue to be generated in the adult hippocampus of the human brain. The answer to this direct question can drastically change the way we think cognitive processes occur in the human brain and how we deal with a huge volume of constantly changing information.
